# The Potential Indirect Impact of GLP-1 Receptor Agonists in the Management of Fibromyalgia

**DOI:** 10.3390/jcm15093330

**Published:** 2026-04-27

**Authors:** Nicole Quodling, Frederick R. Carrick, Norman Hoffman, Monèm Jemni

**Affiliations:** 1 The Carrick Institute, Cape Canaveral, FL 32920, USA; nicquod@gmail.com (N.Q.); drfrcarrick@post.harvard.edu (F.R.C.);; 2 Coelevate Chiropractic, Adelaide, SA 5081, Australia; 3 Burnett School of Biomedical Sciences, University of Central Florida, Orlando, FL 32827, USA; 4 MGH Institute of Health Professions, Boston, MA 02129, USA; 5 Centre for Mental Health Research in association with University of Cambridge, Cambridge CB3 9AJ, UK; 6 College of Medicine, University of Central Florida, Orlando, FL 32827, USA; 7 Faculty of Physical Education, Ningbo University, Ningbo 315211, China

**Keywords:** diabetes, fibromyalgia, GLP-1RA, obesity, pain

## Abstract

**Background/Objectives:** Fibromyalgia (FM) syndrome is characterised by constant and pervasive musculoskeletal pain and may be comorbid with obesity. Glucagon Peptide-1 Receptor Agonists (GLP-1RAs) are relatively new pharmacotherapies developed for the treatment of type 2 diabetes mellitus (T2DM) and have been repurposed for the treatment of obesity. In addition to their well-established impact on glucose balance, new evidence indicates that GLP-1RAs may have anti-inflammatory properties beyond glycaemic regulation. The use of GLP-1RAs has been proposed to modulate the central pain pathways in patients with FM; however, few studies have directly evaluated their effects on central pain. Hence, the purpose of this study is to review the relationship between FM and obesity and to explore the potential role of GLP-1RAs in the management of FM. **Methods:** A literature search was conducted across four databases—PubMed/Medline, Cochrane, Google Scholar, and PEDro—up to May 2025. The literature was sparse, and no formal evaluation process was performed; however, papers were excluded if they failed to address either FM or GLP-1RAs. The key characteristics of each study were extracted and summarised in table form to enable efficient narrative synthesis. **Results:** Of the 56 included studies, 24 were preclinical reviews, 16 were clinical reviews, 8 examined preclinical animal models, and only 8 focused on human data, limited to retrospective analyses of data and self-reporting. There is some evidence that GLP-1RAs may reduce neuronal excitability, inhibit pain signalling, and decrease inflammation. **Conclusions:** However, no clinical trials directly evaluating GLP-1RAs in FM were identified, and therefore no conclusions can be drawn regarding clinical efficacy in FM, including in patients with comorbid obesity.

## 1. Introduction

Fibromyalgia (FM) syndrome is characterised by constant and pervasive musculoskeletal pain but encompasses a broad spectrum of clinical manifestations, including hypersensitivity, fatigue, cognitive, sleep, and mood disorders [1,2,3]. Additionally, FM may be comorbid with certain diseases, including rheumatic diseases, infections, and neurological or psychiatric conditions; type 2 diabetes (T2DM) [1]; and obesity [3]. The relationship between obesity and pain may be bidirectional [4]; obesity may be a consequence of reduced physical activity due to pain and fatigue [5] or result from the consumption of more sweet and fatty foods, which is a behaviour associated with pain, a phenomenon termed ingestion analgesia [6].

Nociplastic pain, including FM, is usually treated with first-line treatments including antidepressants, gabapentinoids, and opioids [7,8,9,10]. These drugs may cause adverse effects, including somnolence, dizziness, fatigue, nausea [9], and obesity [4], further complicating the bidirectional relationship between obesity and FM [2,4,11]. Furthermore, current therapeutics are insufficient for many patients, underscoring the need for new therapies [8].

Where FM is comorbid with obesity, creating an energy deficit is the cornerstone of effective weight management [11]. Lifestyle modification, specifically, reducing daily caloric intake and increasing physical activity, is the first recommendation for weight management [2,12], supplemented, if necessary, by pharmacological and, in justified cases, surgical treatment [13,14]. However, patients with chronic musculoskeletal pain and functional limitation may find it more challenging to exercise and maintain calorie restriction [5,15]. Bariatric surgery is currently superior in its effects regarding both weight loss and its maintenance, as well as accompanying comorbidities [2,16,17,18]. The dramatic weight loss observed in bariatric surgery patients has been shown to significantly decrease median pain scores in patients with FM [19] and has a positive effect on peripheral neuropathy in animal studies [20]. However, bariatric surgical procedures have inherent risks and adverse effects on bone metabolism, leading to an increased risk of fractures [2,19,21]. The key to achieving adequate pain relief relies on management of a comprehensive, multifactorial, and personalised treatment [4].

Glucagon Peptide-1 Receptor Agonists (GLP-1RAs) are a category of medications that are frequently used to treat T2DM and mimic the function of the naturally occurring hormone GLP-1, predominantly released by intestinal L-cells upon food consumption [1,6]. The uptake of these relatively new medications has been rapid, with a greater proportion of users being female (73.85%) [22]. In addition to their well-established impact on glucose balance, new evidence indicates that GLP-1RAs may have anti-inflammatory properties beyond glycaemic regulation, with GLP-1Ras demonstrating some benefits in alleviating pain in animal models [6,7,23]. Studies in preclinical animal models of diabetic neuropathy indicate that these compounds may target multiple pathways, modulating oxidative stress, ion channels, neurotransmitter systems, and cytokine and inflammatory pathways [8,24].

Given the significance of FM and the current gaps in our understanding of its mechanisms, it is crucial to investigate further clinically relevant factors associated with this syndrome, including obesity. Furthermore, it is crucial to establish whether the analgesic effects of GLP-1RAs demonstrated in preclinical studies of diabetic neuropathy extend to other pain syndromes in human populations. The use of GLP-1RAs has been proposed to modulate the central pain pathways in patients with FM; however, few studies have directly evaluated their effects on central pain. Thus, with the lack of available clinical studies, the purpose of this study is to perform a narrative review to investigate the relationship between FM and obesity and explore the potential role of GLP-1RAs in the management of FM. As a narrative review, no clinical efficacy is implied.

## 2. Materials and Methods

This review was registered with PROSPERO and conducted in accordance with PRISMA guidelines (Supplementary Materials) [25]. A preliminary search of PubMed/Medline and the Cochrane Database of Systematic Reviews was conducted, and no current or underway systematic reviews or scoping reviews on the topic were identified. Then, a literature search was conducted across four databases—PubMed/Medline, Cochrane, Google Scholar, and PEDro—up to May 2025. The search combined the terms “fibromyalgia” OR “chronic widespread pain” with “GLP-1 receptor agonist”, “GLP-1RA”, “Semaglutide,” “Liraglutide,” OR “Tirzepatide”. Boolean operators and filters for peer-reviewed studies published in English were applied where applicable.

For the search strategy, two search domains based on the PICOS principle were identified: population: patients with fibromyalgia and/or chronic widespread pain; and intervention: GLP-1RAs. Studies were excluded if they did not address GLP-1RAs or pain. Although the review was prospectively registered and a systematic search strategy was conducted in accordance with PRISMA guidelines, no studies met the predefined PICOS criteria for formal inclusion. Consequently, the manuscript presents a narrative synthesis informed by the systematic search results

After removing duplicates, 204 studies remained for screening. After retrieval and screening by title and abstract, 58 citations were collated and uploaded into EndNote 2025. Two studies were removed, as they did not refer to GLP-1RAs, pain, or obesity-related disorders in the text.

**Figure 1:**
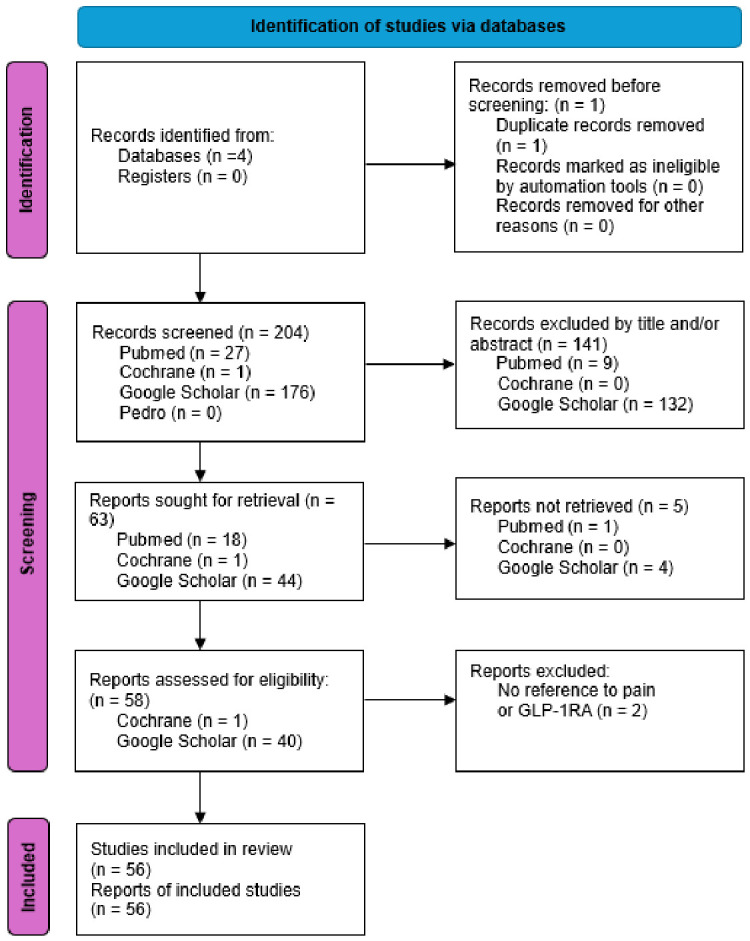
PRISMA 2020 flow diagram [25].

Fifty-six papers were retained for narrative synthesis after screening, as outlined in the PRISMA diagram (Figure 1). Of the 56 included studies, 24 were preclinical reviews, 16 were clinical reviews, 8 examined preclinical animal models and only 8 focused on human data, limited to retrospective analyses and self-reporting. Animal studies were included, despite the difficulty with translation to human populations, in order to summarise large bodies of evidence and identify knowledge gaps as a precursor to considering clinical trials. As this study is a review of previously published research rather than a clinical trial or experimental investigation, the risk of bias was assessed independently by at least two reviewers, and discrepancies were resolved through discussion or adjudication by a third reviewer. The key characteristics of each study were extracted and summarised in a table form to enable efficient narrative synthesis. The narrative nature of this review does not imply clinical efficacy.

## 3. Results

Preclinical reviews, clinical reviews, and the methodologies and findings of preclinical and clinical studies are shown in Table 1. The table summarises literature considered relevant to the hypothesis, including preclinical studies, reviews, and indirect human evidence from adjacent populations. They do not constitute evidence of demonstrated efficacy of GLP-1RAs in FM patients. Note that each section includes a specific summary that highlights the take-home message.

### 3.1. The Relationship Between Fibromyalgia and Obesity

Twenty-four per cent of FM patients are obese, with 5% body weight loss related to mild improvement of FM symptoms [44]. The contribution of obesity to the pathophysiology of FM may involve reduced movement quality, increased joint load, and postural changes [2,4,9,14,45,54], as well as increased systemic inflammation through heightened innate immune cell activation [14]. Adipose tissue has been historically considered amorphous; however, it plays a significant role in immune function and serves as a source of numerous hormones, making it one of the largest endocrine glands in the body [11,19,54]. Hypertrophic adipocytes have been observed to accumulate macrophages in preclinical studies, which can promote inflammation and heighten insulin resistance [9,12]. These cells release substantial amounts of pro-inflammatory cytokines, such as tumour necrosis factor (TNF), interleukin-1 (IL-1), and interleukin-6 (IL-6) [2,5]. Increased cytokines lead to higher serum inflammatory markers, including C-reactive protein (CRP) and erythrocyte sedimentation rate (ESR), as well as elevated oestrogen levels and adipokines [38,44], and neurotransmitter imbalances [33]. Obesity has been linked to significant alterations in dopaminergic pathways, especially in females who show distinct connectivity changes in networks related to salience and emotional regulation [46]. Dopamine dysregulation is a hallmark of FM [1] and is observed in T2DM [3,7]. Dysregulation of the interaction between gut microbial products and the host endocrine system has also been shown to indirectly alter hormonal responses, further exacerbating the severity of obesity, pro-inflammatory responses, and impaired metabolic regulation [12,16]. Altered endocrine function may be associated with high oestrogen [44], leading to greater pain sensitivity [52].

Obesity is significantly associated with the development of metabolic disorders, including T2DM and dyslipidaemia [15,17,46]. T2DM is characterised by hyperglycaemia associated with defective insulin secretion or utilisation [32] and is among the most significant causes of disability worldwide [3]. Complications of T2DM may occur, including diabetic retinopathy [31,32], peripheral neuropathy [2,8,9], and major depressive disorder, often comorbid with FM [3]. The development of neuropathic and depressive complications in T2DM is a complex process that remains incompletely understood [3]; however, it seems to be strongly related to the toxic effects derived from hyperglycaemia and hyperlipidaemia [24,38]: hyperglycaemia can trigger inflammation, oxidative stress, and mitochondrial dysfunction, ultimately inducing neuronal injury [9,10,30,31], while hyperlipidaemia can contribute to the release of pro-inflammatory cytokines by adipocytes [2,5,12,44]. The consequent oxidative stress and inflammation have been considered major contributors to the progression of T2DM and its complications [9,24,38]. The initial phase of neurodegeneration involves the demyelination or remyelination of small fibres, leading to early deterioration and loss of C and Aδ fibres [9,40]. In hyperglycaemic conditions, microglia, astrocytes, and immune cells have been observed to be activated in the spinal cord of animal subjects, contributing to the initiation and maintenance of nociplastic pain by releasing pro-inflammatory cytokines [1,9,24].

Oxidative stress exacerbates inflammation and impacts the function of ion channels, further contributing to pain [30]. Neuropathic pain associated with T2DM also shows a central component, with studies indicating alterations in functional connectivity within the central nervous system (CNS) [10]. Insulin has been shown to directly inhibit serotonergic activity in the dorsal raphe nucleus and to modulate dopamine release, thereby influencing reward behaviour [3], which is often compromised in FM [1].

The primary alterations noted in FM include dysfunctions in monoaminergic neurotransmission, resulting in increases in excitatory neurotransmitters, decreases in serotonin and noradrenaline within the descending antinociceptive pathway, and dysregulation of dopamine and endogenous opioids [1]. The interconnectedness of obesity and FM suggests that the treatment of each condition may depend on the management of another [4].

Multiple proposed pathways, including modulation of oxidative stress, ion channels, neurotransmitter systems, and cytokine and inflammatory pathways, are common to obesity and FM and are summarised in Table 2.

As can be seen, FM and obesity share a high burden of overlapping comorbidities, including chronic pain, fatigue, mood and sleep disorders, cognitive impairment, and peripheral neuropathy, suggesting convergent clinical phenotypes. Both conditions are characterised by shared pathological mechanisms involving chronic low-grade inflammation and neuroinflammation, oxidative stress, mitochondrial dysfunction, insulin resistance, monoaminergic dysregulation, and microbiome alterations, all of which may contribute to central sensitisation and impaired pain modulation.

### 3.2. How Are Obesity and Comorbid FM Being Addressed?

Weight loss appears to reduce serum inflammatory markers, thereby improving pain severity and reducing medication use [44]. Regular exercise leads to an adaptation in antioxidant capacity and effectively reduces inflammation, expressed by the reduction in pro-inflammatory mediators, such as CRP, IL-6, and TNF, and the increase in anti-inflammatory cytokines such as interleukin-4 (IL-4), IL-10, and adiponectin [45], with an increase in mitochondrial function [46]. Caloric restriction-induced weight loss is associated with improvements in symptoms of small fibre neuropathy in animal studies [20].

Weight reduction is challenging, in part, because of metabolic adaptations and hormonal changes that favour weight regain [2,16]. Neuromodulation has emerged as a promising strategy for addressing obesity by targeting neural circuits that govern eating behaviours and metabolic regulation [46]. Photobiomodulation therapy (PBM) may reduce the risk of metabolic complications and comorbidities associated with obesity by reducing fat accumulation, lowering serum lipid levels, and enhancing the production of anti-inflammatory mediators. However, PBM does not demonstrate clear advantages in reducing body fat percentage or insulin levels [21].

### 3.3. Medication

Pharmacotherapy is considered in addition to lifestyle modification when a non-pharmacological approach is not sufficiently effective [2]. Metformin is a first-line pharmacological treatment for most patients with T2DM [38]. Since metformin is effective in modifying T2DM, it is likely to reduce diabetic comorbidities and has been shown to improve the symptomatology of FM [27]. Emerging evidence suggests that metformin exhibits both direct and indirect antioxidant and anti-inflammatory properties, as evidenced by its reduction in several pro-inflammatory cytokines, although these effects have not been fully clarified [38]. Although metformin can activate neurons and microglia to the same extent in male and female rats, it does not affect nerve injury-induced neuropathic pain in female rats [7]. The options for treating T2DM and obesity have recently expanded with the introduction of GLP-1RAs, which modulate the incretin hormonal system [2,16].

### 3.4. What Are GLP-1 Receptor Agonists?

GLP-1 is synthesised and secreted by intestinal enteroendocrine L-cells [6,13,23,29,30,31,38,43,49,50] and specific neurons within the nucleus of the nucleus tractus solitarius (NTS) [8,18,28,49]. GLP-1 is released by the intestinal tract following food intake, and induces the secretion of insulin, thereby modulating blood glucose [31]. GLP-1 acts on the GLP-1 receptor (GLP-1R), which is found on pancreatic islets, as well as throughout the gastrointestinal tract, the vagus nerve, the hypothalamus, the brainstem [1,23], dorsal root ganglia, and the spinal cord [49,50]. Expression of Glucagon-Like Peptide-1 Receptors (GLP-1Rs) has been demonstrated in the pancreas, stomach, and several important brain areas, including the dorsal vagal complex of the brainstem and the hypothalamus [1,49,53]. GLP-1 plays a crucial role in stimulating insulin release in response to elevated glucose levels [6,8,11,38] and suppressing glucagon release [18,24,29,34,43,49]. GLP-1 also reduces gastric emptying and suppresses orexigenic pathways [11,13,18,22,30,49] through both peripheral and central mechanisms [11,28]. However, GLP-1 has a short circulatory half-life of 1–2 min due to rapid enzymatic degradation by dipeptidyl peptidase IV (DPP-4) [6,49]. To overcome this limitation, GLP-1 analogues have undergone extensive structural modifications aiming to reduce degradation while retaining their pharmacological functions [28,49].

GLP-1RAs are a class of medications that mimic the actions of endogenous GLP-1 [29]. GLP-1RAs have been shown to reduce mortality in patients with T2DM [6,8,13,23,26,30,42] andGLP-1RAs stimulate pancreatic β-cells to release insulin only when blood glucose levels are elevated, improving glycaemic control while lowering the risk of hypoglycaemia, reducing glucagon output, and decreasing hepatic glucose production [2,13]. have transformed the management of T2DM and its complications, including diabetic neuropathy [8,27], primarily due to their ability to regulate blood glucose levels and induce significant weight loss [11,28,29].

The use of GLP-1RAs has expanded beyond T2DM management to obesity treatment [13,18,20,23,26]. The Food and Drug Administration (FDA) has approved several GLP-1RAs, separated into short-acting and long-acting formulations [22,28]. Semaglutide is a GLP-1RA similar in structure to the native GLP-1, but it has a half-life of 7 days, compared to the 2 to 3 min of the native hormone [11]. Semaglutide is administered as either a weekly injection or a daily oral tablet [13]. Liraglutide is an intermediate-acting GLP1-RA that shares 97% sequence homology with human GLP-1 [24] and has a half-life of approximately 13 h. It is administered once daily, independently of meals [43]. Tirzepatide combines two intestinal incretin agonists: GLP-1 and gastric inhibitory peptide (GIP) [8,11]. Like semaglutide, tirzepatide is administered by injection once weekly [15]. The dual mechanism of tirzepatide promotes insulin secretion [8] but provides better glycaemic control and weight loss effects than semaglutide [22].

Apart from their glycaemic-lowering effect, GLP-1RAs are considered pharmacological options for treating the complications of T2DM, including peripheral neuropathy [8,31]. This interest has extended to the effects of GLP-1 and its receptor (GLP-1R) signalling on chronic and neuropathic pain [5,26,28,30,34,39,50]. Multiple chronic pain models have evidenced the impacts of GLP-1RAs, demonstrating neuroprotective effects by modulating neuroinflammation and pathways related to neuronal survival [29,31]. Furthermore, GLP-1RAs may alleviate symptoms commonly associated with FM, including hyperalgesia and allodynia [30], depression, and cognitive impairment [3,24]. Thus, preclinical studies suggest the action of GLP-1RAs may have a role to play in alleviating the symptoms of FM, through direct or indirect effects [1,7].

### 3.5. Proposed Mechanisms of Action of GLP-1RAs on Pain

#### 3.5.1. Reduced Inflammation

Weight loss associated with GLP-1RAs leads to reductions in inflammatory markers associated with excess adiposity [5,55], potentially resulting in analgesic effects [26]. However, improvements in pain related to weight loss may stem from reduced body mass [45] rather than decreases in inflammatory markers associated with a smaller volume of adipose tissue [28]. GLP-1RAs may also play a direct role in modulating inflammatory responses by targeting GLP-1R in immune cells [29], thereby reducing microglial cell activation [30,50]. GLP-1RAs decrease pro-inflammatory cytokines [28,57] and induce expression of anti-inflammatory cytokines in preclinical chronic pain models, potentially reducing the sensitisation of nociceptors [24,31]. Moreover, microglial GLP-1R signalling interrupts nociceptive glutamatergic transmission mediated by cytokine expression, desensitising nociceptive circuits from peripheral receptors to the brain mediated by the spinal cord [49,51]. Neuroinflammation has been associated with central sensitisation in preclinical models of FM [1,7,30].

#### 3.5.2. Reduced Oxidative Stress

GLP-1RAs have been shown to reduce oxidative stress and improve neuronal function in preclinical studies [20,28,29] by decreasing ROS production [8] and inducing antioxidant enzymes [38]. Additionally, GLP-1RA-based treatments have been shown to protect against ROS-induced mitochondrial dysfunction in preclinical studies [24,27,30]. A failure of mitochondrial function is known to reduce adenosine triphosphate (ATP) production, leading to increased intracellular calcium levels and increased ROS formation, which contribute to neuronal degeneration and apoptosis, thereby contributing to pain [30]. There is evidence of ROS formation in animal models of FM [7].

#### 3.5.3. Regulation of Cell Proliferation

GLP-1R expressed in the central nervous system (CNS) may regulate cell proliferation, neuronal excitability, and synaptic plasticity [50]. Animal studies have demonstrated that Schwann cells and oligodendrocytes express GLP-1R [29]. GLP-1RAs have demonstrated the ability to stimulate oligodendrocyte progenitor cell differentiation in animal studies, leading to enhanced myelination [20] and reduced cell apoptosis [24,31]. GLP-1RAs may facilitate peripheral nerve regeneration, providing a dual therapeutic effect that combines pain relief with nerve repair [30].

#### 3.5.4. Direct CNS Effects

GLP-1RAs have been shown to cross the blood–brain barrier (BBB) and activate GLP-1R in the CNS [30]. Animal studies have found GLP-1Rs in brain regions such as the NTS, ventrolateral medulla [34], hypothalamus, cortex, hippocampus [18,28,49], cerebellum, brainstem, and microglia of the spinal cord, areas shown to regulate nociception [50]. In inflammatory pain circumstances, preclinical studies have demonstrated robust antinociceptive effects via spinal endorphin release and microglial modulation [1,6,28] and increased firing rates in afferent vagal nerves [34]. Further research is needed to investigate the levels and distribution of GLP-1 in the spinal cord, especially in the dorsal horn [49].

#### 3.5.5. Neuroendocrine Modulation

GLP-1 pathways may modulate dopaminergic midbrain pathways that regulate appetite, reward, learning, memory, and executive control [12,46]. Preclinical studies have shown that deficiency or dysregulation of GLP-1R has been linked to symptoms consistent with FM, including behavioural, mood, and cognitive disturbances [28]. The modulation of central pain pathways via GLP-1RA-based therapies has been proposed in patient groups suffering from FM [30]. Beyond dopaminergic dysfunction, FM and metabolic disorders such as T2DM share disturbances across multiple neurochemical systems, including serotonergic and noradrenergic descending pain pathways [3,18,33], glutamatergic excitatory transmission involved in central sensitization [6,49], and hypothalamic–pituitary–adrenal axis regulation [3,12]. However, the effects of GLP-1 on the peripheral nervous system and its relationship with pain modulation remain largely unexplored [6].

#### 3.5.6. Transient Receptor Potential Channel Activation

A potential candidate for GLP-1RA intervention is the transient receptor potential vanilloid 1 (TRPV1) channel, which plays a crucial role in heat and pain perception and is expressed in sensory neurons. In chronic pain states, TRPV1 channels are upregulated in nociceptive neurons, leading to hyperalgesia, allodynia, and altered temperature perception [52]. GLP-1RAs have effectively inhibited TRPV1 activation in mouse dorsal root ganglion neurons, reducing pain behaviours [6,49]. While testosterone inhibits TRPV1 expression, oestrogens increase TRPV1 expression [52].

**Figure 2:**
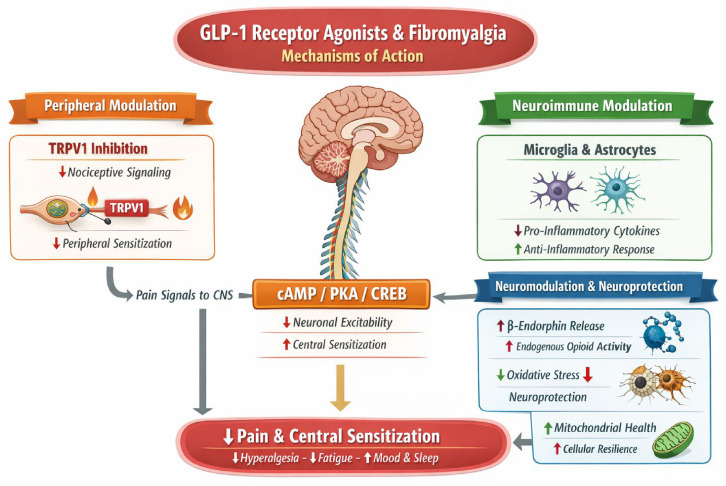
Possible mechanisms by which GLP-1RAs might influence FM. GLP-1Ras may reduce pro-inflammatory cytokines and modulate microglia and astrocytes toward a more homeostatic, anti-inflammatory state. Evidence is predominantly preclinical, with clinical relevance yet to be established.

The possible neurological and analgesic effects of GLP-1RAs are summarised in Figure 2. It is a conceptual model derived from preclinical, review-level, and indirect clinical literature. It is intended to illustrate biological plausibility only and should not be interpreted as evidence of demonstrated therapeutic benefit of GLP-1RAs in FM patients.

### 3.6. Known Adverse Effects

Many of the studies discussed did not mention safety parameters or any adverse effects of the GLP-1RAs [29]; however, the most common adverse effects are gastrointestinal issues [13,40,41,47,48]; injection site reactions [30]; hypoglycaemia, headache, fatigue, and dizziness [23]; and sarcopenia [14]. Individuals with T2DM who also suffer from sarcopenia often exhibit a significant increase in GLP-1 levels [37]. Less commonly reported but more concerning adverse effects include gallbladder disease [43], pancreatitis [11,13,56], pancreatic cancer [32], and thyroid cancer [22]. Clinical trials only reviewed potential risks among those with a Body Mass Index of more than 30 or 27 with one or more weight-related comorbidities, so off-label effects have not been assessed [23]. The growing use of GLP-1RAs, including their long-term intended and off-label use, prompts the need to investigate possible adverse effects and additional impacts [55].

## 4. Discussion

The objective of this study was to review the relationship between FM and obesity and explore the potential role of GLP-1RAs in the management of FM. The well-established association between obesity and widespread musculoskeletal pain, even in non-weight-bearing sites, underscores the complex interplay between weight status and pain in FM [1,2].

It is tempting to conclude that the neuroprotective effects of GLP-1RAs demonstrated in diabetic neuropathy [24,29,30,31] and in preclinical animal studies of FM [1] may alleviate pain. However, an 18-month randomised controlled trial of GLP-1RAs demonstrated no significant effect on neuropathy, including symptomatology and nerve fibre density when compared to insulin treatment [20].

Although the anti-inflammatory efficacy of GLP-1RAs may reflect reductions in glucose and body weight, direct or indirect engagement of the GLP-1R with the immune system may also contribute [53]. By reducing the expression of pro-inflammatory cytokines and activating anti-inflammatory signalling, GLP-1RA-based therapies may mitigate the inflammatory processes that sustain pain in preclinical models of chronic pain [24,30]. However, clinical benefit is yet to be demonstrated.

There is evidence to support high numbers of mitochondrial reactive oxygen species (ROS) in preclinical models of FM [7]. Low levels of ATP at rest, low phosphorylation potential, reduced total oxidative capacity, and reduced number and size of mitochondria in the skeletal muscle of patients with FM have been identified, contributing to potential muscle fatigue and nociceptor sensitisation [39]. GLP-1RA-based treatments have been shown to protect against ROS-induced mitochondrial dysfunction in animal studies [27,31].

GLP-1 is synthesised within the NTS [8,18,28], with receptors located within the vagus nerve and brainstem [1,23]. GLP-1 pathways may modulate descending monoaminergic midbrain pathways that underlie the regulation of appetite, reward, learning, memory, and executive control, as well as the affective component of pain [12,46], which are often dysregulated in FM patients [1].

TRPV1 channels are upregulated in nociceptive neurons in chronic pain states, lowering stimulation thresholds and increasing pain perception, as reported in hyperalgesia or allodynia. Preliminary findings indicate that GLP-1 binds to and inhibits the activation of TRPV1 channels in sensory neurons, broadening the potential pain modulation mechanisms of GLP-1 [6].

Many GLP-1RAs have already been approved for the treatment of T2DM [28]. GLP-1RAs have also shown potential in managing various proposed pathophysiological mechanisms implicated in the initiation and maintenance of FM, through direct impact on weight loss [19,45] or metabolic improvement of inflammation [29,31], oxidative stress [24,30,36], mitochondrial dysfunction [7,39], and central pain processing [1,35]. However, although GLP-1RAs have a favourable safety profile in T2DM management, their long-term use in non-diabetic neuropathic pain populations requires further evaluation [30]. Furthermore, variability exists in the efficacy and mechanisms of different GLP-1RAs and between genders, complicating protocol standardisation [28]. Despite the female predominance of FM [6,28] and the larger uptake of GLP-1RAs in female patients [17,22], there have been no gender-specific studies, and all rodent studies were performed on male animals. Future research should also focus on optimising GLP-1RAs for CNS targeting and exploring combination therapies for enhanced pain relief [31]. The translation to clinical practice hinges on addressing these critical gaps, including the need for condition and gender-specific, rigorous human trials and long-term safety assessments [28]. Clinical benefits are yet to be demonstrated.

## 5. Conclusions

This study reviewed the relationship between FM and obesity and explored the potential role of GLP-1RAs in the management of FM through weight loss, metabolic improvement, or comorbidity reduction. There are significant unmet needs in the management of FM. GLP-1RAs are a rapidly advancing class of drugs, and they are quickly becoming a popular mediator in the treatment of both obesity and T2DM. However, the available data are limited, and, given the lack of clinical trials, no firm conclusions can be reliably drawn regarding the role of GLP-1RAs in the management of FM, with no demonstrated clinical benefit. The mechanisms and effects on neuropathic pain in preclinical animal models indicate that these compounds may target multiple pathways, modulating oxidative stress, ion channels, neurotransmitter systems, and cytokine and inflammatory pathways with a biologically plausible adjunct in the multidisciplinary management of FM. However, several challenges remain before their widespread application: First, clinical research into adverse effects, understanding potential applications, and optimising dosing strategies is needed to ensure the continued safety of these medications. Second, in obesity-associated conditions, distinguishing the direct analgesic effects of GLP-1RAs from the secondary benefits of weight reduction remains a methodological challenge. Third, rigorous evaluation is essential to determine whether the therapeutic benefits observed in animal studies can be reliably and safely reproduced in human patients across various pain modalities, including FM. Lifestyle intervention remains the cornerstone of obesity management in FM.

## 6. Limitations

These results need to be interpreted with caution. This is an emerging field, and, accordingly, the literature is sparse. The current evidence is largely indirect and derived from preclinical models or adjacent clinical populations, rather than FM-specific trials. The majority of papers were reviews, and preclinical animal studies were included because the evidence base is not sufficiently mature. However, preclinical animal data may not reliably predict the safety and efficacy of an intervention when trialled in humans, and reliance on secondary sources can compound bias or lead to misinterpretation of indirect findings. The search was limited to four major databases (PubMed/Medline, Cochrane, Google Scholar, and PEDro) and English-language publications. Small sample sizes, short-term trials, and inconsistent findings limit most studies, and there was heterogeneity of methods and outcomes, including variations in assessed outcomes and a lack of gender specificity. The limited amount of scientific data rules out drawing firm conclusions about the benefit of GLP-1RAs in obese patients with FM. There is no demonstrated direct clinical benefit for the use of GLP-1RAs in the management of FM.

## 7. Future Directions

Future research will be essential to fully elucidate the long-term efficacy and safety of GLP-1RAs. The adverse effects of GLP-1RAs, particularly with off-label and sustained use, warrant further exploration in clinical studies. These therapies must be robustly trialled in people with FM and obesity to evaluate their efficacy, safety, and cost-effectiveness. Additionally, exploring the interactions between GLP-1RAs and other commonly prescribed therapies could reveal how these treatments synergise to improve clinical outcomes.

Some GLP-1RAs show variable efficacy depending on gender, which limits generalisability, especially since FM shows female predominance, and GLP-1Ras have mainly been used by women.

Further research on this topic in clinical studies would be especially valuable for clinicians in need of a new, multidisciplinary personalised pain therapy.

## Figures and Tables

**Table 1:** Preclinical reviews, clinical reviews, and preclinical for animal and human studies.

Study Nature	Author	Findings	Summary	
**Preclinical Reviews**	Baeza-Flores et al., 2020 [7]	GLP-1RA improves clinical symptoms (pain, fatigue, depression, disturbed sleep, and tender points) in FM.	Preclinical reviews suggest GLP-1RAs may modulate biological pathways relevant to fibromyalgia, including inflammatory signalling, metabolic regulation, and neuroprotective mechanisms involved in central pain processing. However, the current evidence base is limited and largely indirect	
	Bober et al., 2024 [9]	GLP-1RA improves clinical symptoms (pain, fatigue, depression, disturbed sleep, and tender points) in FM.		
	Chiang et al., 2022 [20]	Targeting multiple pathological mechanisms a potential therapeutic intervention rather than solely managing symptoms in terms of neuroprotection and/or antinociception in peripheral neuropathy.		
	Cholerzyńska et al., 2024 [26]	Sleep disorders are prevalent among patients with connective tissue diseases and are influenced by various factors including obesity, inflammation and cytokines.		
	Das et al., 2021 [27]	Liraglutide shown to downregulate inflammasome in preclinical studies in rodent models of non-alcoholic fatty liver disease.		
	Gan et al., 2024 [18]	Exogenous GLP-1 causes increased satiety, reduced food intake, delayed gastric emptying, and improved glucose tolerance. While understanding of the neuroendocrine circuitry regulating appetite has advanced, treatment options remain limited due to incomplete knowledge of these complex pathways.		
	Ghusn et al., 2022 [4]	Obesity, depression and chronic pain often comorbid, recommend avoiding medications that increase body weight.		
	He et al., 2025 [28]	GLP-1RAs show promise as novel therapeutics for pain management by exerting neuroprotective and metabolic regulatory effects.		
	Ispas et al., 2023 [12]	The gut microbiota plays a crucial role in the etiology of obesity and metabolic disorders, influencing energy homeostasis and immune responses and potentially serves as a biomarker for prebiotic interventions.		
	Kaye et al., 2024a [13]	GLP-1RAs manage both obesity and T2DM, offering additional benefits for cardiovascular and kidney health, necessitating further research into broader therapeutic applications.		
	Kaye et al., 2024b [29]	GLP-1RAs may act as neuroprotective agents, improving the management of multiple sclerosis, demonstrating neuroprotective effects and potential for improving axonal regeneration and remyelination.		
	Krupa et al., 2024 [3]	Data suggest a significant role of IR in the clinical presentation, pathophysiology and treatment response in major depressive disorder, indicating a need for further investigation and integration of IR into clinical practice.		
	Kuthati et al., 2025 [30]	GLP-1RAs show promise in the management of neuropathic pain by targeting inflammation, oxidative stress, and mitochondrial dysfunction, but further clinical trials are needed to establish their efficacy and safety.		
	Liu et al., 2024 [31]	GLP-1RA, particularly liraglutide, show potential neuroprotective effects in treating diabetic peripheral neuropathy.		
	Naimi et al., 2017 [32]	Rosemary extracts have demonstrated protective effects against hyperlipidemia and hyperglycemia, suggesting potential therapeutic applications for managing blood glucose levels and T2DM.		
	Norwitz & Naidoo, 2021 [33]	Nutritional strategies can be effective in treating anxiety as a metabolic disease.		
	O’Malley, 2016 [34]	GLP-1 alleviated some IBS symptoms through antispasmodic and pain-relieving properties. GLP-1 increases firing rates in afferent vagal nerves.		
	Pușcașu et al., 2024 [35]	Preclinical studies have identified a range of pharmaceutical drugs and natural compounds that show promise in alleviating vincristine induced peripheral neuropathy, including GLP-1RAs.		
	Røikjer et al., 2026 [10]	Lack of tools for assessment of both large and small fibre integrity in T2DM. Lack of personalised treatment approaches for neuropathic pain. Need for improved diagnostic tools and personalized treatment approaches for painful diabetic peripheral neuropathy.		
	Su et al., 2024 [36]	Inflammatory biomarkers are recommended to be assessed in major depressive disorder, and anti-inflammatory therapy is recommended to be included in the clinical practice guidelines.		
	Syed et al., 2023 [8]	GLP-1RAs show promise as a potential treatment for diabetic peripheral neuropathy, but more research is needed to determine their efficacy and the best patient candidates for these treatments.		
	Tan & Fu, 2024 [37]	GLP-1RA may potentially influence the occurrence and development of sarcopenia.		
	Teodoro et al., 2019 [38]	The neuroprotective action of GLP-1 may be related to improved endothelial function through its anti-inflammatory and antioxidant effects.		
	Warrayat et al., 2025 [39]	GLP-1RAs promote growth and protect pancreatic beta cells from apoptosis and ER stress.		
**Study Nature**	**Author**	**Findings**	**Summary**	
**Clinical Reviews**	Azmi et al., 2019 [40]	Painful diabetic neuropathy significantly impacts quality of life, is often underdiagnosed and inadequately managed, and has no FDA approved disease-modifying therapies currently available.	Clinical reviews suggest obesity and metabolic dysfunction may contribute to FM symptom burden through inflammatory, neuroendocrine, and biomechanical pathways that influence central pain processing and physical function. Although weight management strategies, including lifestyle interventions, pharmacotherapy such as GLP-1RA, and surgical approaches, may indirectly modulate these mechanisms, evidence for direct disease-modifying effects in FM remains limited and requires FM specific investigation.	
	Bohler et al., 2024 [11]	Obesity is a chronic, multifactorial disease that requires a comprehensive approach to management. Creating an energy deficit the core of treatment of obesity. Physical activity important to maintain weight reduction. Medications have a role in reducing food consumption.		
	De Wit et al., 2016 [41]	The placebo response in T2DM treatments varies significantly among different drug classes, with injectable GLP-1RA showing a substantial effect on weight.		
	Ebell & Grad, 2022 [42]	GLP-1RA prevent adverse cardiovascular and renal outcomes in patients with T2DM and also reduce all-cause and cardiovascular mortality.		
	Ellegaard et al., 2025 [43]	The evidence supporting use of GLP-1RAs in treating bile acid diarrhoea is limited. Sporadic case reports about the effects GLP-1-based drugs are emerging.		
	Gallo et al., 2018 [44]	Bariatric surgical weight loss reduces serum inflammatory markers and appears to reduce joint pain and improve physical function.		
	Kozakowski et al., 2023 [2]	Obesity significantly contributes to various musculoskeletal disorders, necessitating comprehensive management that includes lifestyle modifications and, when necessary, pharmacological or surgical interventions.		
	Langworthy et al., 2024 [45]	GLP-1RAs is potentially a disease modifying therapy for osteoarthritis.		
	Lespessailles et al., 2019 [19]	Bariatric surgery can improve outcomes in obese patients with rheumatic disease, but it also carries risks, including increased fracture rates.		
	Liu et al., 2025 [46]	Obesity significantly impacts brain structure and function, necessitating targeted interventions that leverage neuroimaging to develop personalized treatment strategies.		
	Palmer et al., 2021 [47]	GLP-1RA reduce cardiovascular mortality, myocardial infarction, kidney failure, and serious hyperglycaemia and lowers body weight without incurring severe hypoglycaemia.		
	Petrinović et al., 2024 [16]	Bariatric surgery significantly alters the pharmacokinetics of statins, necessitating personalized medication management and continuous monitoring of lipid profiles post-surgery.		
	Sattar et al., 2025 [14]	Obesity significantly exacerbates rheumatic and musculoskeletal diseases, necessitating urgent weight management interventions.		
	Shang et al., 2024 [48]	This review aims to assess the efficacy and safety of pharmacological treatments for weight management in adults with overweight or obesity without T2DM, and to compare these interventions with lifestyle modifications.		
	Siebert et al., 2025 [5]	Weight loss of ≥5% body weight can improve psoriatic arthritis, holding promise for improvements with GLP-1RA.		
	Sun et al., 2025 [21]	PBM may be a safe and effective treatment for obesity, showing significant reductions in weight, BMI, and waist circumference.		
**Study Nature**	**Author**	**Findings**	**Subjects**	**Summary**
**Preclinical Animal Studies**	Go et al., 2024 [6]	GLP-1 derived peptides, demonstrate significant analgesic effects by modulating TRPV1 activity, providing a potential alternative for chronic pain management.	Adult wild-type male C57BL/6 N mice	Preclinical evidence suggests that GLP-1RA may influence mechanisms relevant to FM through modulation of microglial activity, inflammatory signalling, and nociceptive pathways, including TRPV1-related processes and central sensitization. However, these findings are largely derived from animal and experimental models, and their translational relevance to FM pathophysiology and clinical outcomes remains to be established through targeted human studies.
	Gong et al., 2014 [49]	Spinal GLP-1Rs expressed on microglial cells and are upregulated after peripheral nerve injury, contributing to the antinociceptive effects of GLP-1R agonists in pain hypersensitivity.	Wistar rats	
	Jing et al., 2021 [50]	Activation of microglial GLP-1R in the trigeminal nucleus caudalis suppresses central sensitization of chronic migraine.	Male C57BL/6 mice weighing 18–20 g	
	Ma et al., 2021 [51]	GLP-1RA shown to alleviate neuropathic pain by modulating microglial activation and inflammatory responses.	Male Wistar rats	
	Moustafa et al., 2018 [24]	Liraglutide has been shown to ameliorate diabetic peripheral neuropathy in rats by improving blood glucose levels, reducing oxidative stress, and preserving nerve structure.	Male Wistar rats	
	Pietrowicz & Root-Bernstein, 2025 [52]	Capsaicin binds to insulin and ESR1, enhancing estradiol binding and insulin sensitivity, influencing energy metabolism, particularly in women. TRPV1, ESR1 and insulin share significant regions of homology.	Enzyme-linked immunosorbent assay (ELISA)—animal and human cells	
	Shafiek et al., 2025 [1]	Semaglutide shows benefits in a reserpine rat model of FM, demonstrating improvements in sensory and motor behavioural deficits, reduction in inflammation, and neuroprotection.	Male Wistar rats of approximately 3 months age, weighing 150 ± 20 g	
	Wong et al., 2024 [53]	GLP-1RAs reduce inflammation induced by TLR agonists through mechanisms that require central neuronal GLP-1 receptors.	Ten to twelve-week-old male C57BL/6J mice	
**Study Nature**	**Author**	**Findings**	**Subjects**	**Summary**
**Preclinical Huma Studies**	Baser et al., 2024 [54]	GLP-1RA may help prevent osteoarthritis in patients with obesity.	1360 patients with obesity using GLP-1RA, 68.90% female, 71.22 ± 4.51, 39,881 patients with obesity without medication, 55.79% female, 74.08 ± 5.95	Clinical studies suggest that GLP-1RA use in populations with obesity and metabolic disease is associated with improvements in musculoskeletal outcomes and health-related quality of life, alongside recognised safety considerations following wider adoption. However, these observations are indirect, derived from heterogeneous populations and study designs, and cannot be assumed to translate to FM, highlighting the need for prospective, specific studies to clarify clinical relevance, risk to benefit profiles, and underlying mechanisms.
	Hayman et al., 2025 [55]	GLP-1R variants have consistent cardiometabolic effects, but their effects on mental ill health phenotypes are more varied, suggesting that behavioral changes associated with GLP-1RA therapy may not be directly mediated through GLP-1R.	484,833, 54.3% female, (40-69)	
	Ho et al., 2025 [23]	GLP-1RA-related hospitalization utilization increased significantly after semaglutide approval, with unintentional therapeutic errors the primary reason.	1047, 74.2% female, mean age 54	
	Hunter Gibble et al., 2025 [15]	Self-reported health-related quality of life outcomes significantly improved in participants with obesity and T2DM who were treated with tirzepatide compared with placebo.	938, 50.7% female, mean age 54.2	
	Javed et al., 2023 [56]	Liraglutide can cause acute pancreatitis as a rare complication.	Case report, male age 73	
	Lewis et al., 2025 [57]	Semaglutide use associated with reductions in wound healing complications and chronic pain in T2DM related foot ulcers.	6329 DFU patients with semaglutide, 6329 DFU patients without semaglutide, both cohorts 37.9% female, 56.7 ± 11.4	
	Li et al., 2025 [22]	Adverse effects associated with tirzepatide included injection site pain, nausea, injection site haemorrhage, diarrhoea, and vomiting, incorrect doses, off-label use and hypoglycaemia.	8096 tirzepatide adverse effects reported, 73.84% female	
	MacEwan et al., 2021 [17]	<1% of adults eligible for antiobesity medications in 2015–2018 used them, but utilization of newer medications increased significantly between 2015–2016 and 2017–2018.	34 obesity patients using medication, 79.9% female, 46.6, 6310 obesity patients not using medication, 49.5% female, 49.2	

***Abbreviations:** Glucagon Peptide-1 Receptor Agonist (GLP-1RA); Fibromyalgia (FM); Glucagon Peptide-1 (GLP-1); Type 2 Diabetes Mellitus (T2DM); Insulin resistance (IR); Irritable bowel syndrome (IBS); Food and Drug Administration (FDA); Photobiomodulation (PBM); Body Mass Index (BMI); Transient receptor potential vanilloid 1 (TRPV1); Estrogen receptor 1 (ESR1); Toll-like receptor (TLR); Diabetic Foot Ulcer (DFU).*

**Table 2:** Overlap of clinical manifestations and underlying pathophysiology in obesity and fibromyalgia.

	Obesity	Fibromyalgia
**Neuropathy**	2 studies suggest obesity contributes to peripheral nerve disorders [9,20]	1 study suggests FM shares similar mechanisms to peripheral neuropathy [7]
**Pain**	8 studies suggest obesity is associated with chronic pain conditions [2,4,5,9,14,19,27,28]	3 studies suggest chronic widespread pain is a hallmark of FM [1,7,28]
**Fatigue**	2 studies suggest obesity contributes to fatigue [9,14]	4 studies suggest fatigue is a common clinical symptom of FM [2,7,8,47]
**Depression/Mood Disorders**	7 studies suggest obesity contributes to the development of depression [3,4,11,27,31,48,53]	5 studies suggest mood disorders are a common comorbidity of FM [1,2,3,7,47]
**Cognitive Disorders**	1 study suggests high BMI is associated with increased cognitive impairment risk [31]	2 studies suggest cognitive impairment is a common comorbidity of FM [1,47]
**Sleep Disorders**	6 studies suggest obesity contributes to obstructive sleep apnoea [11,17,19,31,45,53]	3 studies suggest sleep disorders are a common comorbidity of FM [1,2,7]
**Mitochondrial Dysfunction**	1 study suggests exercise improves mitochondrial function in obesity [31]	2 studies suggest decreased mitochondrial biogenesis in FM [7,47]
**Oxidative Stress**	1 study suggests oxidative stress is a potential biomarker for obesity [12]	2 studies suggest decreased antioxidant enzyme expression levels in FM [7,35]
**Inflammation**	7 studies suggest chronic low-grade systemic inflammation is associated with obesity [2,9,18,27,31,37,45]	3 studies suggest neuroinflammation mediated by microglia contributes to central sensitisation in FM [1,7,35]
**Dopamine Dysregulation**	5 studies suggest food addiction in obesity is associated with heightened connectivity in dopaminergic pathways [4,12,31,48,53]	1 study suggests dopaminergic dysregulation in FM [1]
**Noradrenaline Dysregulation**	2 studies suggest noradrenergic dysregulation in obesity [4,53]	2 studies suggest decreases in noradrenaline levels at the descending antinociceptive pathway level [1,57]
**Serotonin Dysregulation**	2 studies suggest serotonergic dysregulation in obesity [4,53]	2 studies suggest decreases in serotonin levels at the descending antinociceptive pathway level [1,57]
**Insulin Resistance**	8 studies suggest obesity is associated with IR [2,4,7,9,12,16,21,31]	2 studies suggest a connection between IR and FM [1,7]
**Microbiome Dysfunction**	3 studies suggest obesity is associated with dysbiosis [12,16,19]	1 study suggests digestive dysfunction is associated with FM [47]

*Abbreviations: fibromyalgia (FM); Body Mass Index (BMI); insulin resistance (IR).*

## Data Availability

No new data were created or analyzed in this study.

## Supplementary Materials

The following supporting information can be downloaded at: https://www.mdpi.com/article/10.3390/jcm15093330/s1, PRISMA 2020 Checklist; PRISMA 2020 for Abstracts Checklist.
